# Structural insights into physiological activation and antagonism of melanin-concentrating hormone receptor MCHR1

**DOI:** 10.1038/s41421-024-00754-0

**Published:** 2024-11-30

**Authors:** Xiaofan Ye, Guibing Liu, Xiu Li, Binbin He, Yuyong Tao, Jiasheng Guan, Yuguang Mu, Haiping Liu, Weimin Gong

**Affiliations:** Division of Life Sciences and Medicine, University of Science and Technology of China, Hefei, Anhui China

**Keywords:** Hormone receptors, Cryoelectron microscopy

Dear Editor,

Mammalian melanin-concentrating hormone (MCH) is a cyclic peptide pivotal in regulating various physiological processes, including but not limited to feeding behavior, energy homeostasis, sleep-wake cycles, cognitive functions, emotional states, and nociception^1^. Two G protein-coupled receptors for MCH, MCHR1 and MCHR2, have been identified in humans, with MCHR1 being common to all mammals. Predominantly localized within the central nervous system^2^, MCHR1 mediates the physiological responses to MCH, rendering it a promising therapeutic target for various diseases encompassing obesity, sleep disorders, anxiety disorders, schizophrenia, and Alzheimer’s disease^3–6^. Despite the great importance of comprehending the regulatory mechanisms of MCHR1 activity through its activating or inhibiting ligands, the lack of structural insights persists. Here, we report the cryo-EM structures of both the active-state MCHR1 bound to MCH and G_i1_, and the inactive-state MCHR1 complexed with a selective antagonist, SNAP-94847. Through a combination of structural analysis and functional assays, we delineate the recognition of MCH and SNAP-94847 by MCHR1 and unveil the mechanisms governing receptor activation and antagonism.

To capture the active conformation of MCHR1 in complex with MCH and heterotrimeric G protein, we co-expressed human MCHR1 with the three subunits of G_i1_ in Sf9 insect cells, followed by purification in the presence of synthetic MCH peptide (Supplementary Data. S1). Using cryo-EM, the structure was determined in multiple states, of which the best reached the resolution of 2.61 Å (Fig. 1a; Supplementary Figs. S1, S2 and Table S1). These states are distinguished by variations in the relative orientation of MCHR1 and the G_i1_ heterotrimer (Supplementary Fig. S1b), with two states (T1 and T2) exhibiting tighter receptor‒G protein contacts over the other two (L1 and L2), owing to distinct conformations of the first intracellular loop (ICL1) of MCHR1 (Supplementary Fig. S3a‒c). A recent study also reported the structure of the MCH‒MCHR1‒Gi complex^7^, but only a single state that is similar to the L1 state of our structure. This might be resulted from different strategies for data processing. These structures collectively support the existence of the L1 state (Supplementary Fig. S9a‒c). Notably, upon alignment of the structures by the receptor, with the C-terminal α5 helix of Gα_i1_ almost fixed, a sequential displacement of the αN helix was observed, characterized by a clockwise rotation from the L2 state to the T2 state (Supplementary Fig. S3d). Despite these differences, MCHR1 in all conformations exhibits a fully active state, with nearly identical modes of G-protein coupling. The primary coupling interface is established by the α5 helix of Gα_i1_ and the intracellular cavity of MCHR1 (Supplementary Fig. S3e). Additionally, ICL2 of MCHR1 adopted an ordered short helix conformation, interacting with the αN‒α5 hydrophobic patch (Supplementary Fig. S3f), while ICL3, along with the intracellular terminus of TM6, forms an additional interface with Gα subunit at the β6 strand and the α4‒β6 loop (Supplementary Fig. S3g). Interactions between ICL1 and G protein were less observed previously. Consistently, in the loose conformations, ICL1 exhibits only a loose contact with the surface of Gβ (Supplementary Fig. S3h). However, in the tight conformations, K139, L140, and C143 of ICL1 form close interactions with G_i1_ at the αN‒Gβ interface, with L140 protruding into the crevice between the αN helix and Gβ (Supplementary Fig. S3i). Recently, a study on M_2_R also identified two distinct conformational states of the M_2_R‒G_oA_ complex bound to acetylcholine^8^, compared to the single state of the M_2_R‒G_oA_ complex bound to the potent agonist iperoxo^9^. Structural comparisons reveal differences in the positioning of the Gα subunit between the two states (S1 and S2) of the acetylcholine‒M_2_R‒G_oA_ complex (Supplementary Fig. S10a), with the αN‒α5 angle smaller in the S2 state than in the S1 state. Interestingly, the single state of the iperoxo‒M_2_R‒G_oA_ complex shows an αN‒α5 angle closer to that of the S2 state (Supplementary Fig. S10a), suggesting a potential correlation between agonist efficacy and the conformation of the GPCR‒G protein complex. In our study, the tight conformations of the MCHR1‒G_i1_ complex exhibit a smaller αN‒α5 angle compared to its loose conformations (Supplementary Fig. S10b). Additionally, we identified mutations K139E and K139A, which abolish MCH-induced G_i_ and G_q_ dissociation in HEK293T cells. This finding further underscores the role of ICL-1 in receptor activation and suggests that the balance and transition between these conformations are linked to receptor activation. (Supplementary Figs. S3j, k, S4i).

**Fig. 1:**
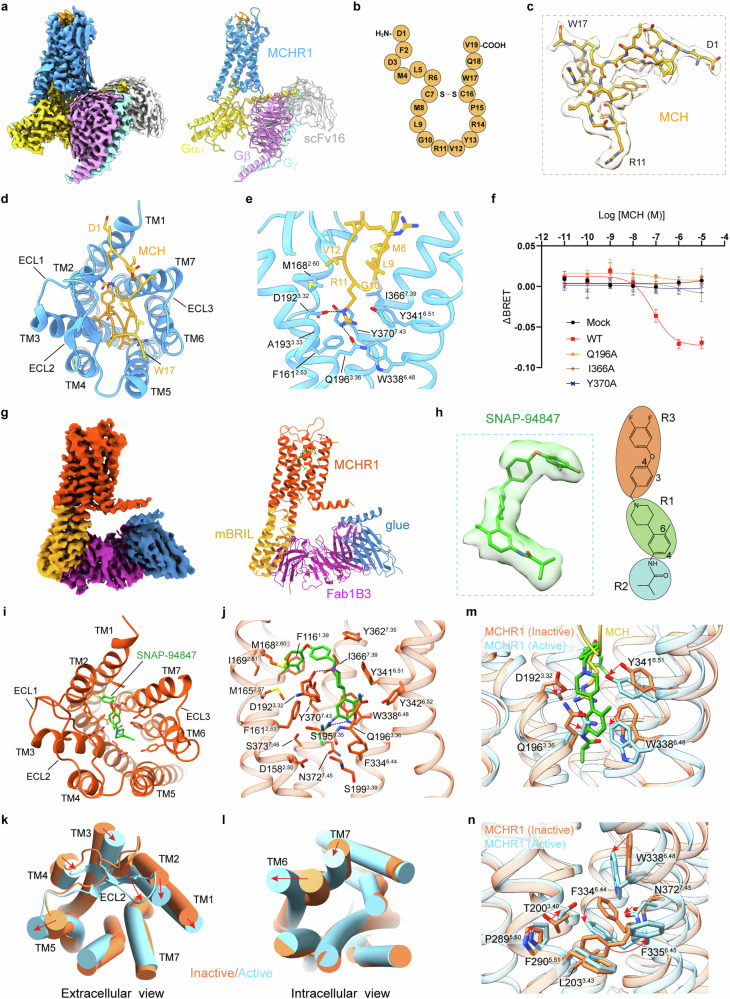
Cryo-EM structures of the MCH‒MCHR1‒G_i1_ complex and antagonist-bound MCHR1. **a** EM map and model of MCH‒MCHR1‒G_i1_ complex in T1 state. **b** Sequence of human MCH. **c** Density map of MCH in T1 state. **d** Extracellular view of MCH‒MCHR1‒G_i1_ complex in T1 state. **e** Interactions between R11 and MCHR1. The hydrogen bonds are depicted as black dashed lines. **f** G_i_-dissociation curves of MCHR1 mutants. Data are shown as means ± SEM from three independent experiments. **g** EM map and model of antagonist-bound MCHR1 complex in the S1 state. **h** Density map and molecular structure of SNAP-94847. **i** Extracellular view of the inactive structure. **j** Interactions between SNAP-94847 and MCHR1. **k** Superposition of active MCHR1 (T1 state) and inactive MCHR1 (S1 state) in the extracellular view. Transmembrane helices (TMs) are shown as cylinders. The movements of TMs are indicated by red arrows. **l** Intracellular view of the superposed structures. **m** Comparison of the ligand-binding pocket. **n** Rearrangement of hydrophobic packing.

Despite the structural variations observed in the MCH‒MCHR1‒G_i1_ complexes, the binding conformation of MCH within MCHR1 remains consistent across different states (Supplementary Fig. S4a). MCH, cyclized by the disulfide bond between C7 and C16 (Fig. 1b), exhibits well-defined density spanning from F2 to W17 in the EM maps, excluding the side chain of W17 (Fig. 1c; Supplementary Fig. S2). Within MCHR1, MCH occupies a large pocket formed by the outer regions of transmembrane helices (TMs) 2, 3, 5, 6, and 7, as well as extracellular loops (ECLs) 1, 2, and 3 (Fig. 1d). The C7‒C16 segment of MCH adopts a short β-hairpin configuration, inserting into the central cavity formed by the transmembrane helices (Supplementary Fig. S4b). Positioned at the distal end of the β-hairpin, R11 extends deeply into the pocket, establishing hydrogen bonds with D192^3.32^ and Q196^3.36^ (superscripts represent Ballesteros-Weinstein nomenclature), while engaging in hydrophobic interactions with F161^2.53^, M168^2.60^, A193^3.33^, W338^6.48^, Y341^6.51^, I366^7.39^, and Y370^7.43^ (Fig. 1e). Mutations of Q196^3.36^, I366^7.39^, and Y370^7.43^ to alanine, without altering the expression levels, markedly impaired MCH-induced G_i_ dissociation in HEK293T cells (Fig. 1f; Supplementary Fig. S4i and Table S2), indicating the crucial role of R11 in MCHR1 activation. On the N-terminal side of the β-hairpin, backbone atoms of MCH establish hydrogen bonds with the sidechains of Q345^6.55^, Q348^6.58^, and Y362^7.35^ (Supplementary Fig. S4c). Mutation of Y362^7.35^ to alanine dramatically compromised MCH-induced G_i_ dissociation (Supplementary Fig. S4f). On the C-terminal side of the β-hairpin, Y13 interacts with the second β-strand of ECL2 via backbone hydrogen bonds, while its sidechain also forms a hydrogen bond with the backbone carbonyl of Q171^2.63^ (Supplementary Fig. S4d). While the β-hairpin engages MCHR1 through extensive interactions with the outer part of the transmembrane domain, the N-terminus of MCH binds within a superficial sub-pocket primarily via interactions with extracellular loops of the receptor (Supplementary Fig. S4e). These observations highlight the critical role of R11 and the β-hairpin structure of MCH in activating MCHR1.

MCHR1 antagonists represent promising therapeutic agents for a spectrum of metabolic disorders and various other diseases. To elucidate the mechanism of MCHR1 antagonism, we endeavored to solve the structure of the inactive-state MCHR1 bound to the selective antagonist SNAP-94847. To this end, we employed a previously established approach^10^, engineering the human MCHR1 by introducing an mBRIL fragment between TM5 and TM6 to replace the ICL3 in a rigid manner, while fusing a K3 helix along with an ALFA tag to the H8 helix. To bridge mBRIL and the H8-K3-ALFA helix, we incorporated specific components, including an anti-BRIL Fab (Fab1B3), and a bivalent glue molecule featured by an N-terminal E3 helix followed by anti-Fab and anti-ALFA nanobodies (NbFab and NbALFA). The engineered MCHR1-mBRIL construct was expressed in Sf9 cells and purified in the presence of SNAP-94847, and then incubated with Fab1B3 and the glue molecule in vitro to generate a complex for structure elucidation via cryo-EM (Supplementary Data. S1). The structure was resolved in two different states at resolutions of 3.33 Å and 3.43 Å, respectively (Fig. 1g; Supplementary Fig. S5 and Table S1). Despite variations in the overall conformation of the entire complex, the conformation of MCHR1 remains largely constant in these two states, exhibiting a typical inactive conformation compared to the active structure (Supplementary Fig. S6a, b). The cryo-EM map of S1 state allowed unambiguous assignment of the majority of MCHR1 (Supplementary Fig. S5g), with additional density observed within the transmembrane helices, attributed to SNAP-94847 (Fig. 1h).

The antagonist SNAP-94847 can be divided into three functional groups, a 4-(2-methylphenyl)piperidine scaffold (R1), an isobutyramido group (R2), and a 4-(3,4-difluorophenoxy)benzyl group (R3) (Fig. 1h), which collectively bind into a hydrophobic pocket surrounded by TMs 1, 2, 3, 6, and 7 (Fig. 1i). Its binding occurs deeper within the TM7 domain compared to MCH, with its R2 group penetrating inward by approximately 7.5 Å relative to R11 of MCH (Supplementary Fig. S6c). We performed a molecular dynamics simulation based on the experimental structure. From the RMSD plot, the protein was shown to be relatively stable over the entire simulation. The maximum RMSD achieved during the simulation at 300 K was only 3.5 Å (Supplementary Fig. S8a). The ligand was found to be unstable when using the docked conformation from the cryo-EM as the ligand and had a conformational change before the first 50 ns (Supplementary Fig. S8b). Despite the conformational changes, throughout the entire simulation, the ligand was lodged at the ligand binding site. The binding of the R1 and R2 groups of SNAP-94847 to MCHR1 seem to be more stable than that of the R3 group, which is consistent with the observation that the R3 group shows higher diversity in SNAP-94847-like ligands^11,12^. The majority of the R1 group establishes tight hydrophobic interactions with Q196^3.36^, F334^6.44^, W338^6.48^, Y341^6.51^, Y342^6.52^, and Y370^7.43^, with the tertiary amine moiety anchored by an ion‒ion interaction with D192^3.32^ (Fig. 1j; Supplementary Fig. S6d). Meanwhile, the R2 group is nestled within a compact sub-pocket deep inside the 7TM domain, comprised of D158^2.50^, F161^2.53^, S195^3.35^, S199^3.39^, F334^6.44^, W338^6.48^, N372^7.45^, and S373^7.46^. This interaction is further reinforced by a hydrogen bond between the carbonyl oxygen of the R2 group and W338^6.48^ (Supplementary Fig. S6e). Notably, the isopropyl moiety at the anilide position has been reported to dramatically enhance affinity^12^, likely attributable to its favorable accommodation within the small sub-pocket. The R3 group engages in hydrophobic interactions with F116^1.39^, M165^2.57^, M168^2.60^, I169^2.61^, Y362^7.35^, I366^7.39^, and Y370^7.43^ (Supplementary Fig. S6f). In this part, an observed preference for 4-aryloxybenzyl analogues over 3-aryloxybenzyl^12^ can be explained by an optimal steric match with the sub-pocket that accommodates the R3 group. Moreover, small electron-withdrawing groups at the end of the R3 group have been associated with favorable MCHR1 affinities^12^, likely due to interactions with F116^1.39^ (Supplementary Fig. S6g‒j and Table S3). These collective effects make SNAP-94847 a high-affinity antagonist of MCHR1.

By elucidating the structures of both the agonist-bound active and antagonist-bound inactive states of MCHR1, we have gained valuable insights into the mechanisms of receptor activation and antagonism. Superimposed structures exhibit substantial differences in both extracellular and intracellular regions. Binding of MCH induces a contraction of the orthosteric ligand-binding pocket, characterized by a remarkable inward shift at the extracellular termini of TMs 2, 3, and 4, as well as ECL2 (Fig. 1k). Intracellularly, a notable outward movement of TM6, coupled with a subtle inward shift of TM7, can be observed, which facilitates receptor coupling with the G protein (Fig. 1l). Within the ligand-binding pocket, binding of MCH promotes a downward displacement of W338^6.48^ (Fig. 1m), accompanied by re-packing of T200^3.40^, L203^3.43^, P289^5.50^, F290^5.51^, F334^6.44^, F335^6.45^, and N372^7.45^, thereby initiating the outward movement of TM6 at the cytoplasmic end (Fig. 1n). In contrast, antagonist occupation restricts the conformation. Specifically, while the tertiary amine of SNAP-94847 is anchored by D192^3.32^, the methylphenyl group at the C4 position of the piperidine group tightly packs against W^6.48^ of MCHR1, preventing the conformational changes of W^6.48^ and nearby residues, thus blocking MCHR1 activation. Docking analysis using the inactive structure suggests that a range of MCHR1 antagonists may share a common mechanism of action (Supplementary Fig. S7).

In summary, our study elucidates the molecular basis for hormone and small-molecule antagonist recognition, activation, G-protein coupling, and inhibition of the melanin-concentrating hormone receptor MCHR1. First, this study enriches our understanding of how GPCRs can recognize different types of ligands, in this case the endogenous peptide and the non-peptide antagonist. Second, by providing both the active and inactive structures of MCHR1, this study offers new insights into GPCR activation, especially regarding the rearrangement of hydrophobic packing at the activation core of the receptor, including a phenylalanine flip during activation. Moreover, this study also provides more clues for understanding of conformational equilibrium and transitions of G-protein-engaging GPCRs. Despite challenges in drug discovery attributed to frequent side effects, MCHR1 remains an appealing therapeutic target for metabolic disorders and various pathological conditions. The antagonist-bound structure presents an unprecedented opportunity for the identification of novel compounds targeting MCHR1 through computational methods, thereby expanding the repertoire of MCHR1 antagonists, potentially yielding new chemotypes that offer solutions to current challenges. Furthermore, the insights regarding MCHR1 signaling also lay a groundwork for investigating the physiological and pathophysiological roles of MCHR1.

## Supplementary information

Supplementary figures

Supplementary Data S1

Supplementary tables
