# Supplementary material for: Structural insights into physiological activation and antagonism of melanin-concentrating hormone receptor MCHR1
Source: Cell Discov. 2024 Nov 30;10:124. doi: 10.1038/s41421-024-00754-0 (PMC11608246; doi:10.1038/s41421-024-00754-0)
Supplement: Supplementary file 1 — Supplementary figures [file 41421_2024_754_MOESM1_ESM.pdf]

Supplementary Figures for

Structural insights into physiological activation and antagonism of melanin-concentrating hormone receptor MCHR1

Xiaofan Ye, Guibing Liu, Xiu Li, Binbin He, Yuyong Tao, Jiasheng Guan, Yuguang Mu, Haiping Liu, Weimin Gong

Table of Contents

Supplementary Figures 2

Supplementary Fig. S1 | Structure determination of the MCH-MCHR1-G_{i1} complex by cryo-EM....................3

Supplementary Fig. S2 | Local resolution, density maps of MCH-MCHR1-G_{i1} structures.......4

Supplementary Fig. S3 | Differences of MCH-MCHR1-G_{i1} structures. 5

Supplementary Fig. S4 | Structural comparison and functional data for the active structure....7

Supplementary Fig. S5 | Structure determination of antagonist-bound MCHR1 by cryo-EM..8

Supplementary Fig. S6 | Structural comparison and functional data for the inactive structure. 10

Supplementary Fig. S7 | Docking results of MCHR1 antagonists. 12

Supplementary Fig. S8 | RMSD plot of both systems across the 400ns molecular dynamics simulation. 13

Supplementary Fig. S9 | Comparison of MCH-MCHR1-Gi1 complex. 14

Supplementary Fig. S10 | Comparison of the orientation of Gα. 15

Supplementary Figures

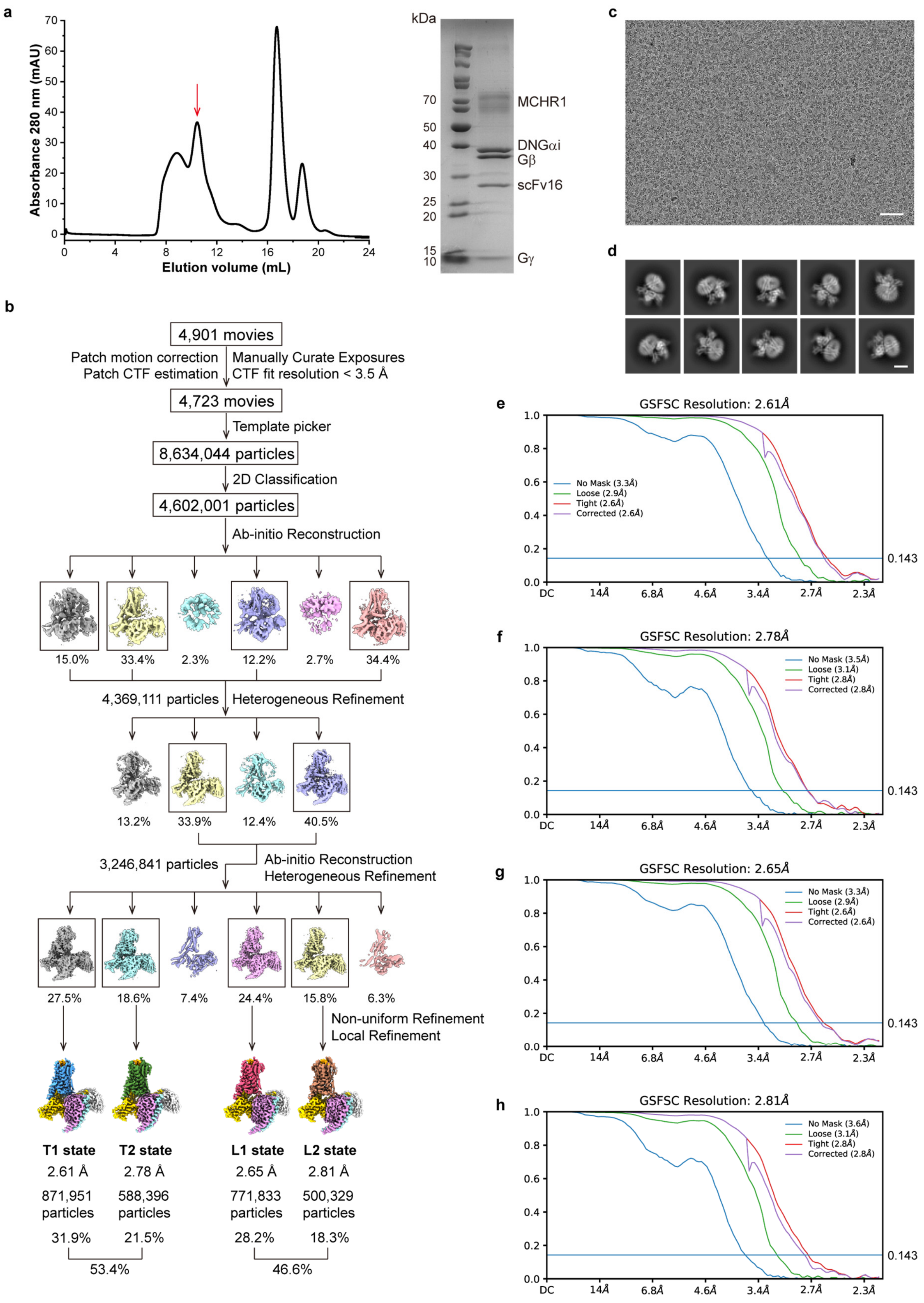

Supplementary Fig. S1 | Structure determination of the MCH-MCHR1-G_{i1} complex by cryo-EM.

a, Size-exclusion chromatography (SEC) and SDS-PAGE (stained by Coomassie blue) profiles of the MCH-bound MCHR1-G_{i1} complex. **b**, Processing workflow of cryo-EM data. **c**, Representative micrograph. **d**, Representative 2D averages. **e-h**, Gold-standard FSC curves for EM maps of T1 state, T2 state, L1 state, and L2 state, respectively.

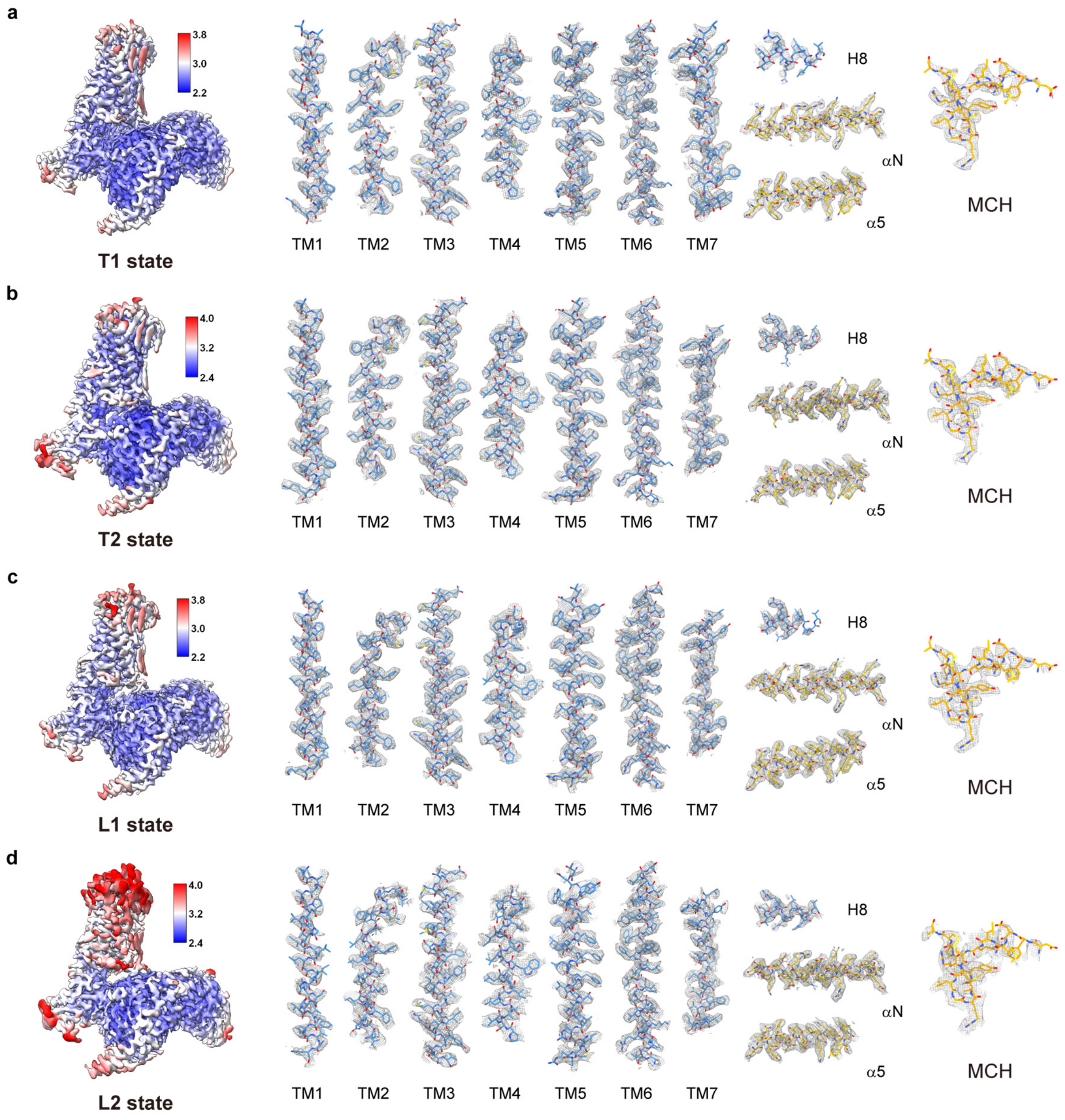

Supplementary Fig. S2 | Local resolution, density maps of MCH-MCHR1-G_{i1} structures.

a-d, Local resolution of EM maps and density maps of transmembrane helices (TM1-TM7), H8, αN helix, α5 helix and MCH. EM maps and density maps are displayed at the contour level of 0.40, 0.33, 0.35, and 0.23 for T1 state, T2 state, L1 state, and L2 state, respectively.

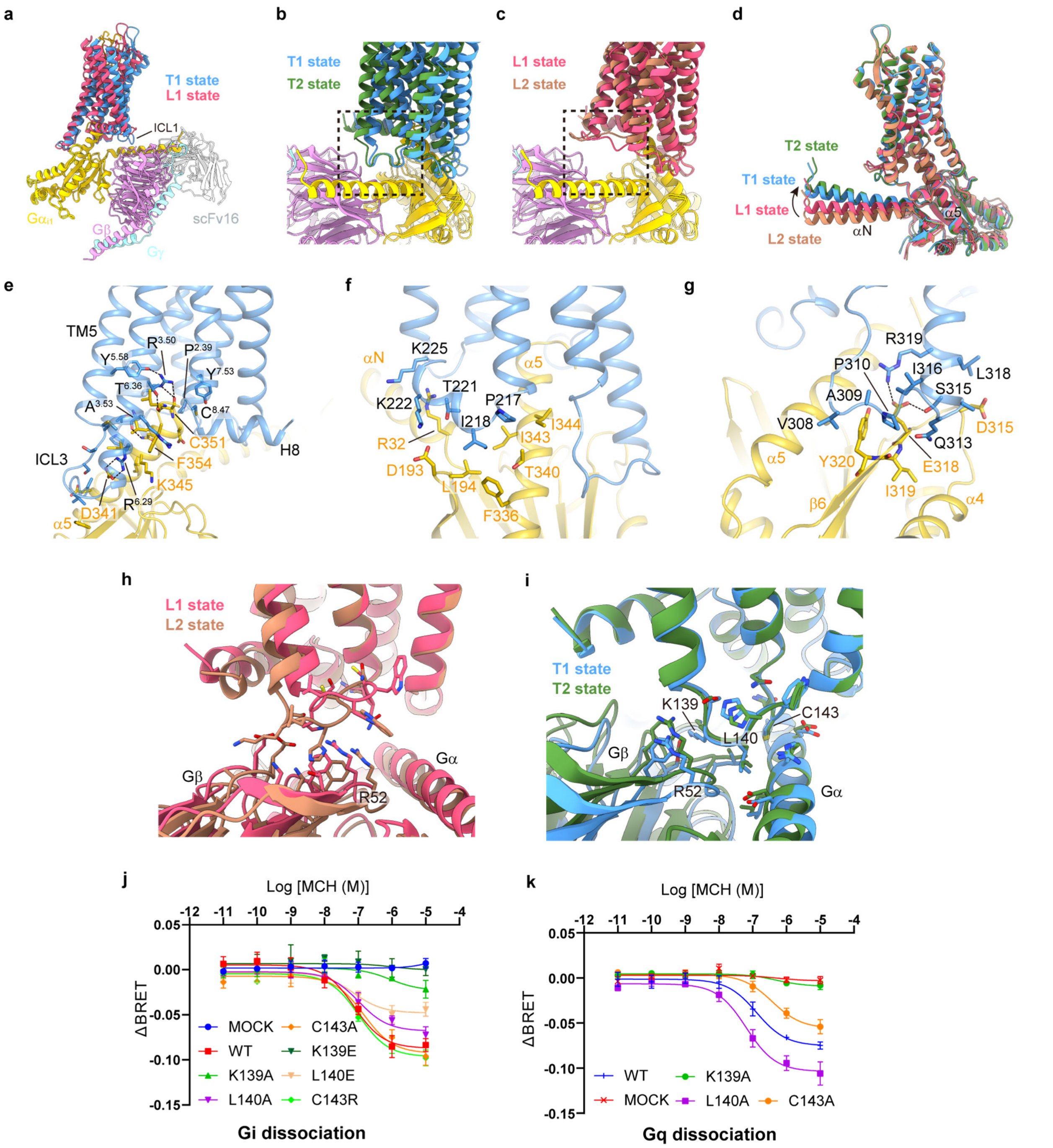

Supplementary Fig. S3 | Differences of MCH-MCHR1-G_{i1} structures.

a, Superposition of T1 state and L1 state (aligned by Gβ). **b**-**c**, The contact between ICL1 and Gi1 in the tight conformers (**b**) or the loose conformers (**c**). **d**, The rotation of αN helix in 4 states (aligned by MCHR1). **e**, Interactions between MCHR1 and the α5 helix of $G\alpha_{i1}$. The hydrogen bonds are depicted as black dashed lines. **f**, Interactions between ICL2 of MCHR1 and the αN-α5 hydrophobic patch. **g**, Interactions between ICL3 of MCHR1 and $G\alpha_{i1}$. **h**, The contact between ICL1 and G_{i1} in the loose states. **i**, The contact between ICL1 and G_{i1} in the tight states. **j**, Effects of ICL1 mutations determined by G_i-dissociation assay. Data are shown as means ± SEM from three independent experiments. **k**, Effects of ICL1 mutations determined by G_q-dissociation assay. Data are shown as

means ± SEM from three independent experiments.

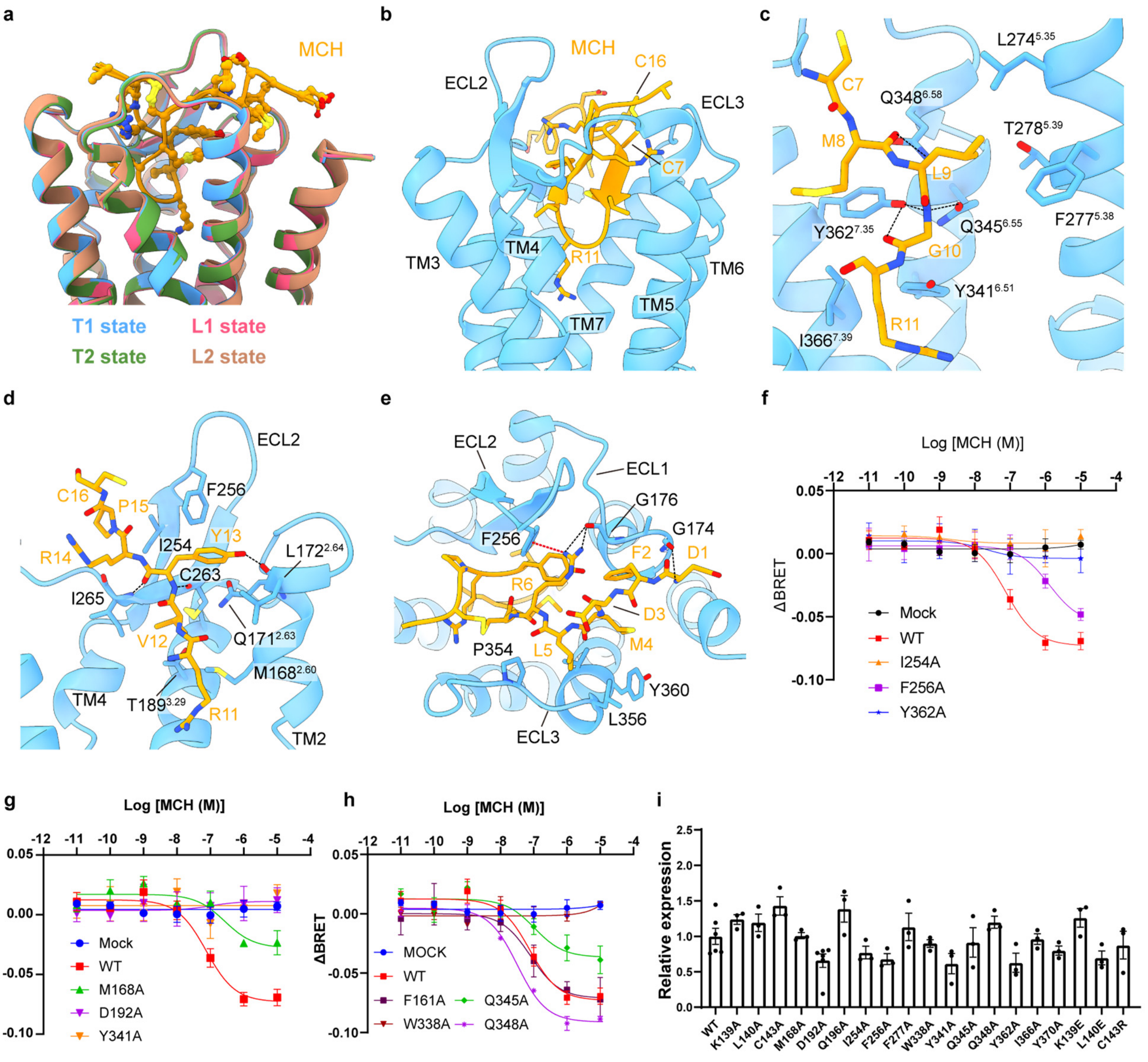

Supplementary Fig. S4 | Structural comparison and functional data for the active structure.

a, Superposition of different conformers of the MCH-MCHR1-G_{i1} complex. **b**, The β-hairpin of MCH and the binding pocket. **c**, Interactions between the N-terminal fragment of the β-hairpin and MCHR1. The hydrogen bonds are depicted as black dashed lines. **d**, Interactions between the C-terminal fragment of the β-hairpin and MCHR1. **e**, Interactions between the N-terminus of MCH and MCHR1. The cation-π interaction between R6 and F256 is depicted as a red dashed line. **f**-**h**, G_i-dissociation curves of MCHR1 mutants. Data are shown as means ± SEM from three independent experiments. **i**, Relative cell-surface expression of MCHR1 mutants in HEK293T cells. Data are shown as means ± SEM from three or six independent experiments.

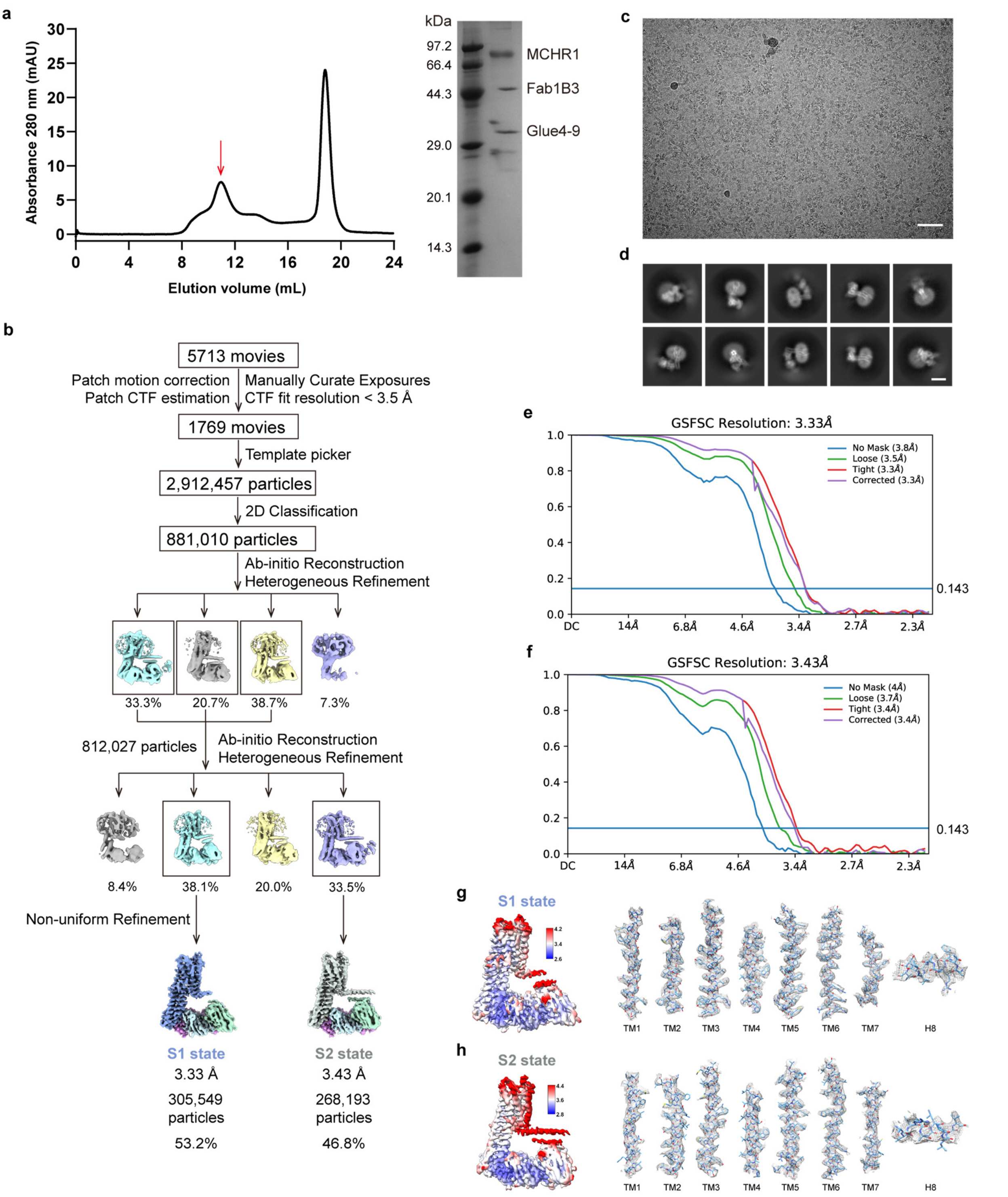

Supplementary Fig. S5 | Structure determination of antagonist-bound MCHR1 by cryo-EM.

a, Size-exclusion chromatography (SEC) and SDS-PAGE (stained by Coomassie blue) profiles of the MCHR1-Fab1B3-Glue complex. **b**, Processing workflow of cryo-EM data. **c**, Representative micrograph. **d**, Representative 2D averages. **e**-**f**, Gold-standard FSC curves for EM maps of S1 state and S2 state, respectively. **g**-**h**, Local resolution of EM maps and density maps of transmembrane helices (TM1-TM7) and H8. Local-resolution EM maps are displayed at the contour level of 0.55

and 0.45 for S1 state and S2 state, respectively. Density maps are displayed at the contour level of 0.45 and 0.40 for S1 state and S2 state, respectively.

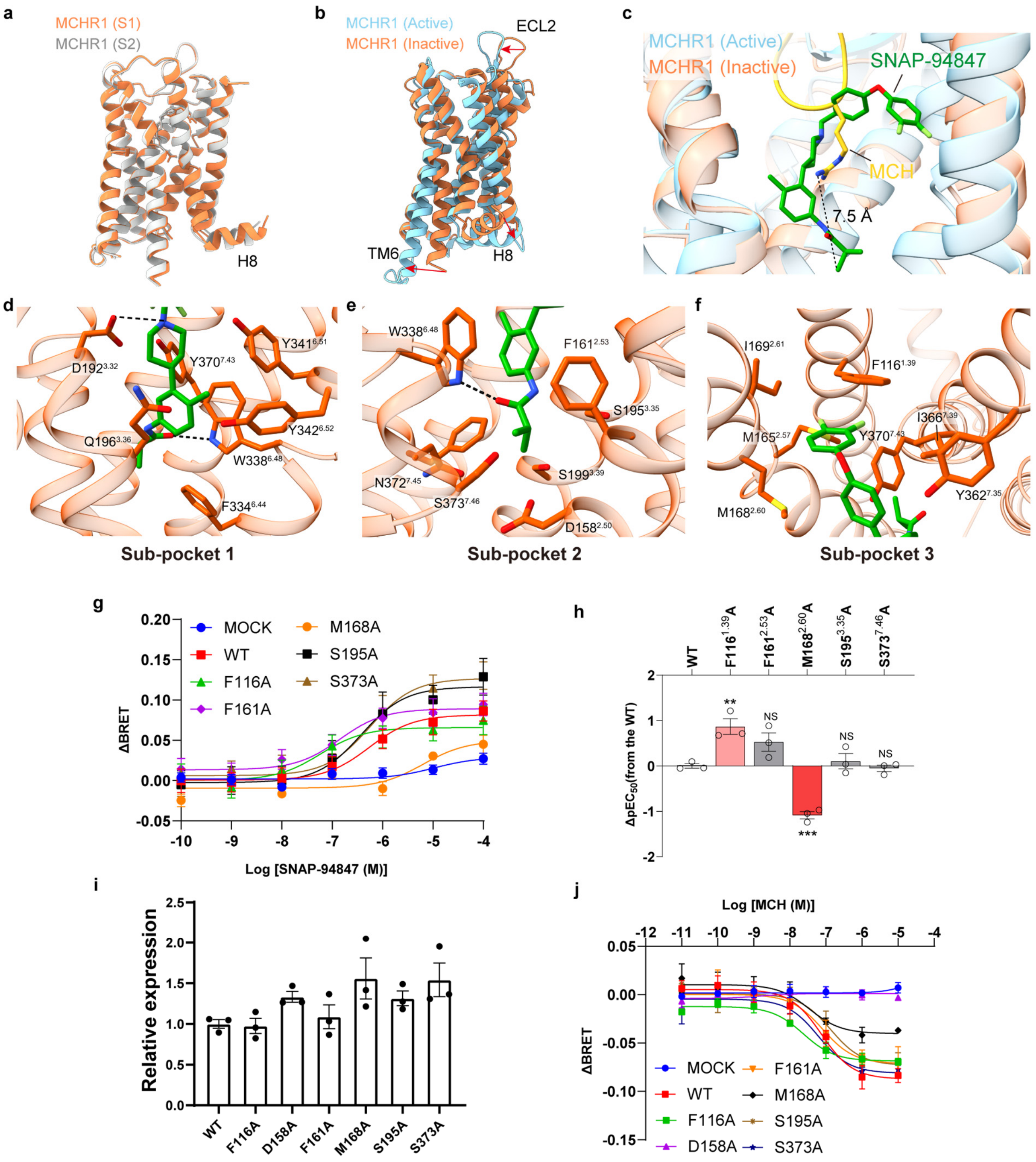

Supplementary Fig. S6 | Structural comparison and functional data for the inactive structure.

a, Comparison of S1 state and S2 state of antagonist-bound MCHR1. **b**, Superposition of the active (T1 state) and the inactive (S1 state) structures of MCHR1. **c**, Comparison of the binding pockets for MCH and SNAP-79847. **d**, Interactions between the R1 group of SNAP-94847 and MCHR1. The ionic interactions and hydrogen bonds are depicted as black dashed lines. **e**, Interactions between the R2 group of SNAP-94847 and MCHR1. The hydrogen bonds are depicted as black dashed lines. **f**, Interactions between the R3 group of SNAP-94847 and MCHR1. **g**, G_i-dissociation curves of MCHR1 mutants in response to SNAP-94847. Data are shown as means ± SEM from three independent experiments. **h**, G_i-dissociation assay results of MCHR1 mutants in response to

different concentrations of SNAP-94847 with 10 μM MCH. ΔpEC_{50} of each mutant is compared to WT using one-way ANOVA with Dunnett's multiple comparisons. $^{**}P < 0.01$, $^{***}P < 0.001$. NS, no significant difference. **i**, Relative cell-surface expression of MCHR1 mutants in HEK293T cells. Data are shown as means ± SEM from three independent experiments. **j**, G_i-dissociation curves of MCHR1 mutants in response to MCH. Data are shown as means ± SEM from three independent experiments.

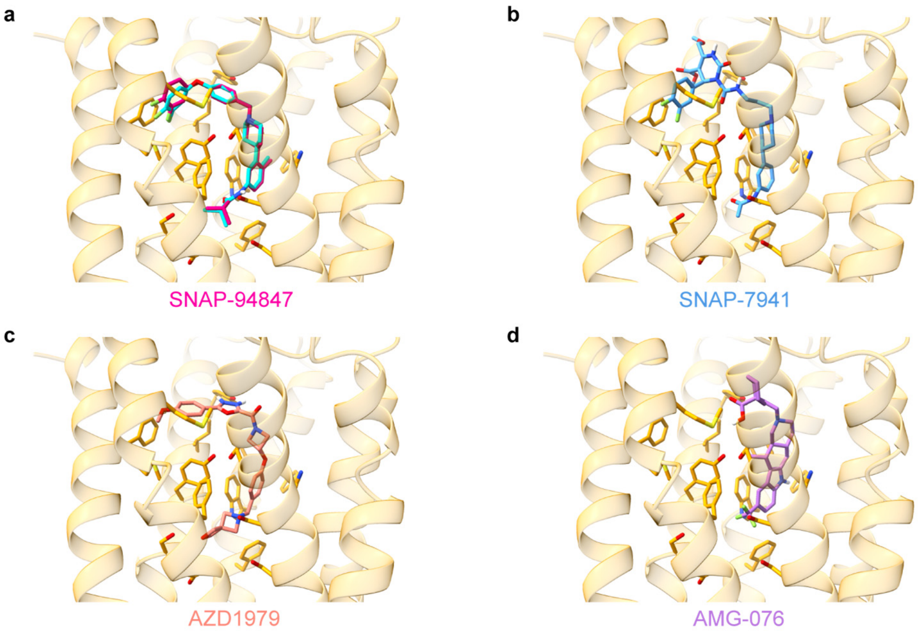

Supplementary Fig. S7 | Docking results of MCHR1 antagonists.

a, Superposition of SNAP-94847 in the cryo-EM structure (cyan) and the docked pose (magenta). **b-d**, Docked poses of other MCHR1 antagonists.

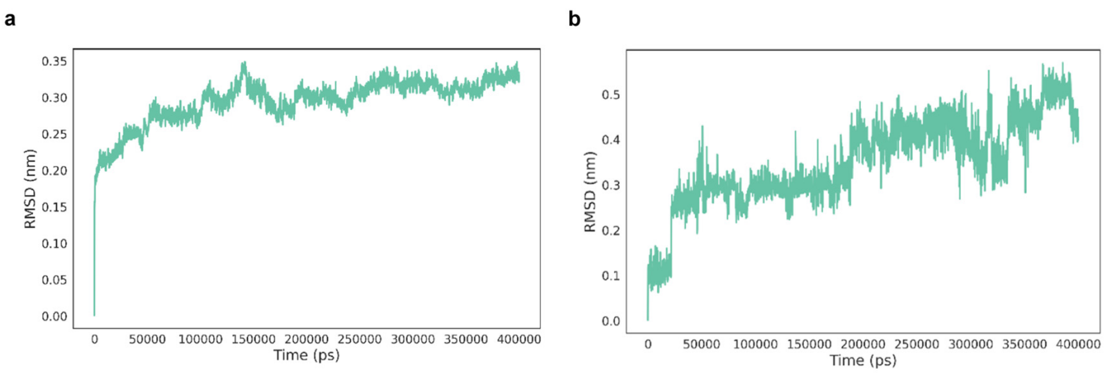

Supplementary Fig. S8 | RMSD plot of both systems across the 400ns molecular dynamics simulation.

a-b, RMSD of (a) MCHR1 protein without BRIL and C-tag sequences and (b) SNAP-94847 ligand.

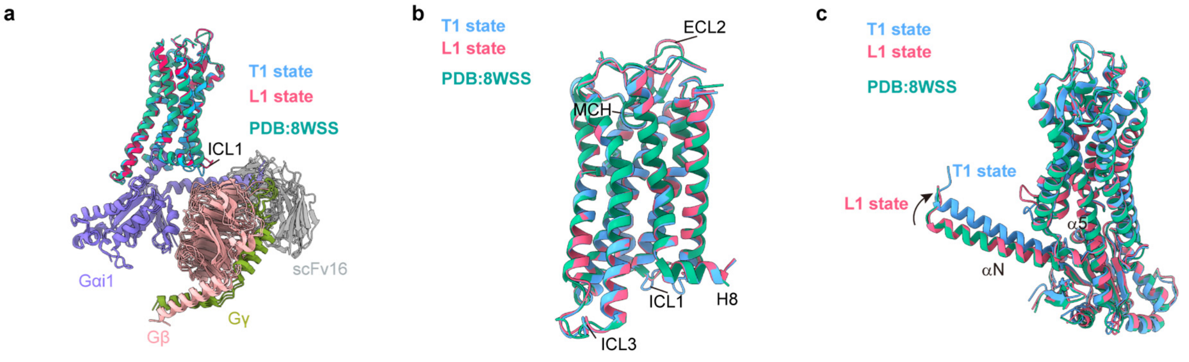

Supplementary Fig. S9 | Comparison of MCH-MCHR1-Gi1 complex.

a, Superposition of three different states of MCH-MCHR1-Gi1 structures: L1 state, T1 state and recent reported state (PDB ID: 8WSS), aligned by receptor. **b,** Superposition of the T1 state, L1 state and recent reported state of MCHR1 (PDB ID: 8WSS). **c,** Superposition of the T1 state, L1 state and recent reported state of MCHR1-Gi1 (PDB ID: 8WSS).

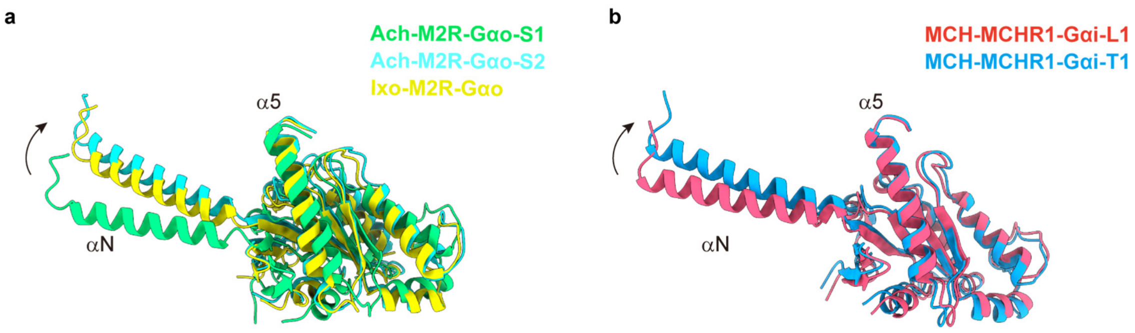

Supplementary Fig. S10 | Comparison of the orientation of Gα.

a, Comparison of the orientation of Gαo in ACh-bound S1 (PDB ID: 7T8X), ACh-bound S2 (PDB ID: 7T90), and Ixo-bound (PDB ID: 6OIK) states. **b**, Comparison of the orientation of Gαi in MCH-bound L1 and T1 states.
