# Supplementary material for: Structural insights into physiological activation and antagonism of melanin-concentrating hormone receptor MCHR1
Source: Cell Discov. 2024 Nov 30;10:124. doi: 10.1038/s41421-024-00754-0 (PMC11608246; doi:10.1038/s41421-024-00754-0)
Supplement: Supplementary file 2 — Supplementary Data S1 [file 41421_2024_754_MOESM2_ESM.pdf]

Supplementary Information for

Structural insights into physiological activation and antagonism of melanin-concentrating hormone receptor MCHR1

Xiaofan Ye, Guibing Liu, Xiu Li, Binbin He, Yuyong Tao, Jiasheng Guan, Yuguang Mu, Haiping Liu, Weimin Gong

Table of Contents

Supplementary Data S1 2

Construct generation 2
Protein purification 2
Cryo-EM sample preparation and data acquisition 4
Cryo-EM data processing 4
Model building and validation 5
G protein-dissociation assay 5
Cell-surface expression analysis 6
Molecular dynamics (MD) simulations 6
Molecular docking 7
Data availability 7

References 8

Supplementary Data S1

Construct generation

For structure determination of the MCH-MCHR1-G_{i1} complex, the wild-type human melanin-concentrating hormone receptor 1 (MCHR1) with a truncated C-terminus (the last 26 amino acids were truncated) was synthesized and constructed into a modified pFastBac1 vector containing a bovine prolactin signal peptide followed by a FLAG tag and an 8× His tag at the N-terminus for purification. To facilitate protein expression, MCHR1 was fused with an N-terminal fragment of β_2AR (BN). A NanoBiT strategy was applied as previously described[1]. An LgBiT subunit was fused with a 17-amino-acid linker (HMGSSGGGGSGGGGSSG) at the C-terminus of the receptor. Human $G\alpha_{i1}$ with four dominant-negative mutations (DN$G\alpha_{i1}$)[2], S47N, G203A, E245A, and A326S, was cloned into the pFastBac1 vector. Human $G\beta_1$ with an N-terminal 6× His tag and a C-terminal HiBiT subunit connected by a 15-amino-acid linker (GSSGGGGSGGGGSSG), and human $G\gamma_2$ were cloned into the pFastBac-Dual vector. Additionally, a single-chain Fab fragment (scFv16) was utilized to stabilize the complex[3]. Coding sequence of the antibody fragment scFv16 with a GP67 signal peptide at the N-terminus and a TEV cleavage site followed by an 8× His tag at the C-terminus was constructed into the pFastBac1 vector.

For structure determination of the antagonist-bond MCHR1, a previously described strategy was applied[4]. To fuse mBRIL to MCHR1 in a rigid fashion, the active-state structure of MCHR1 was aligned with the previous β_2AR-mBRIL construct. Appropriate residues were introduced or deleted to obtain a continuous helix for TM5 and TM6. After determining the junction sequences, AlphaFold was used again to predict the resulting sequence to confirm whether a rigid fusion was formed. For the H8 fusion, a similar strategy was used and the coding sequence was constructed into the pFastBac1 vector. For Fab1B3 and the 4-9 glue molecule, the coding sequences were cloned into the pET-22b(+) vector with an N-terminal pelB signal peptide and a C-terminal 6× His tag.

For functional assays, wild-type MCHR1 was cloned into the pcDNA3.1(+) vector before mutations were introduced individually. All constructs were verified by DNA sequencing.

Protein purification

Expression and purification of scFv16 were conducted as previously described[3]. Briefly, scFv16 was expressed in Tni (HiFive) insect cells and purified by Ni resin. The C-terminal His tag was removed by TEV protease. Proteins were loaded onto a Superdex 200 Increase 100/300 GL column (GE Healthcare) and the correct fractions were pooled, concentrated, flash-frozen and stored at -80 °C before use.

For MCH-MCHR1-G_{i1} complex, MCHR1, DN$G\alpha_{i1}$, $G\beta_1$, and $G\gamma_2$ were co-expressed in Sf9 insect cells using the Bac-to-Bac system (Invitrogen). Cells were infected with three types of viruses encoding MCHR1, DN$G\alpha_{i1}$, $G\beta_1\gamma_2$ at the ratio of 3:2:2 at the density of 2.5×10^6 cells/mL and cultured at 27 °C for 48 h. Cells were collected by centrifugation, flash-frozen and stored at -80 °C

before use. For the purification of MCHR1-G_{i1} complex, cell pellets were thawed in lysis buffer containing 20 mM HEPES pH 7.5, 50 mM NaCl, 10 mM $MgCl_2$, 5 mM $CaCl_2$, 2.5 μg/mL leupeptin, 300 μg/mL benzamidine, 25 mU/mL Apyrase (New England Biolabs), and 100 μM TCEP at room temperature for 2 h. For MCH-bound complex, MCH peptide (synthesized by Sangon Biotech) was added into the lysis buffer at the final concentration of 2 μM and kept at 1 μM in all the following steps. After centrifugation at 30,700 g for 30 min, the cell membranes were resuspended and solubilized in buffer containing 20 mM HEPES pH 7.5, 100 mM NaCl, 0.5% (w/v) lauryl maltose neopentylglycol (LMNG, Anatrace), 0.1% (w/v) cholesteryl hemisuccinate (CHS, Anatrace), 10% (v/v) glycerol, 10 mM $MgCl_2$, 5 mM $CaCl_2$, 12.5 mU/mL Apyrase, 2.5 μg/mL leupeptin, 300 μg/mL benzamidine, and 100 μM TCEP for 2 h at 4 °C. The supernatant was collected by centrifugation at 38,900 g for 45 min and then incubated with Ni resin at 4 °C for 2 h. After loaded onto a gravity column, the resin was washed with 20 column volumes of washing buffer containing 20 mM HEPES pH 7.5, 100 mM NaCl, 0.05% (w/v) LMNG, 0.01% (w/v) CHS, 2.5 μg/mL leupeptin, 300 μg/mL benzamidine, 100 μM TCEP, and 20 mM imidazole. Proteins were eluted with the same buffer plus 400 mM imidazole. The eluate was supplemented with 2 mM $CaCl_2$ before incubated with anti-FLAG M1 antibody resin overnight at 4 °C. The FLAG antibody resin was washed with 10 column volumes of washing buffer plus 2 mM $CaCl_2$. The complex was eluted with same buffer containing 5 mM EDTA and 200 μg/mL FLAG peptide. Purified scFv16 was added to the eluate at a 1.3:1 molar ratio. Finally, the complex was purified by a Superdex 200 10/300 column (GE Healthcare) equilibrated with buffer containing 20 mM HEPES pH 7.5, 100 mM NaCl, 0.00075% (w/v) LMNG, 0.00025% (w/v) GDN, 0.00015% (w/v) CHS, and 100 μM TCEP. The peak fractions containing monomeric complexes were pooled and concentrated for EM studies.

For Fab1B3 and the glue molecule, the plasmids were transformed into *E.coli* BL21 (DE3) cells, and cells were grown at 37 °C in LB medium supplemented with 50 μg/mL ampicillin. Cells were induced by the addition of 1 mM IPTG and incubated for 24 h at 16 °C. Cells were collected and disrupted in buffer containing 20 mM HEPES pH 7.5 and 150 mM NaCl. Both Fab1B3 and the glue molecule were purified by Ni-affinity chromatography. Unwanted proteins were removed with wash buffer (20 mM HEPES pH 7.5, 150 mM NaCl, and 20 mM imidazole), and the target protein was eluted with wash buffer supplemented with 300 mM imidazole. The eluate was concentrated to 20 mg/ml using a 10 kDa molecular weight cutoff concentrator (Millipore) for the assembly of complexes.

For antagonist-bond MCHR1, the chimeric MCHR1-mBRIL was expressed in Sf9 insect cells using the Bac-to-Bac system. Cells were infected with virus encoding MCHR1-mBRIL at the density of 2.5×10^6 cells/mL and cultured at 27 °C for 48 h. Cells were collected by centrifugation, flash-frozen and stored at -80 °C before use. Cell pellets were thawed in lysis buffer containing 10 mM HEPES pH 7.5, 0.5mM EDTA, 2.5 μg/mL leupeptin, 150 μg/mL benzamidine at room temperature for 2 h. SNAP-94847 (MedChemExpress) was added into the lysis buffer at the final concentration of 1 μM and kept at 1 μM in all the following steps. Then, excess purified Fab1B3 and the glue molecule were added. After centrifugation at 30,700 g for 30 min, the cell membranes were resuspended and solubilized in buffer containing 20 mM HEPES pH 7.5, 100 mM NaCl, 1% (w/v) LMNG, 0.2% (w/v) CHS, 10% (v/v) glycerol, 2.5 μg/mL leupeptin, 300 μg/mL benzamidine, and

100 μM TCEP for 2 h at 4 °C. The supernatant was collected by centrifugation at 38,900 g for 60 min and then incubated with Ni resin at 4 °C for 2 h. After loaded onto a gravity column, the resin was washed with 20 column volumes of washing buffer containing 20 mM HEPES pH 7.5, 150 mM NaCl, 0.05% (w/v) LMNG, 0.01% (w/v) CHS, 2.5 μg/mL leupeptin, 300 μg/mL benzamidine, 100 μM TCEP, and 20 mM imidazole. Proteins were eluted with the same buffer plus 400 mM imidazole. The eluate was supplemented with 5 mM $CaCl_2$ before incubated with anti-FLAG M1 antibody resin overnight at 4 °C. The resin was washed with 10 column volumes of washing buffer plus 2 mM $CaCl_2$. The complex was eluted with same buffer containing 5 mM EDTA and 200 μg/mL FLAG peptide. Finally, the complex was purified by a Superdex 200 10/300 column equilibrated with buffer containing 20 mM HEPES pH 7.5, 150 mM NaCl, 0.00075% (w/v) LMNG, 0.00025% (w/v) GDN, 0.00015% (w/v) CHS, and 100 μM TCEP. The peak fractions containing monomeric complexes were pooled and concentrated for EM studies.

Cryo-EM sample preparation and data acquisition

An aliquot of 3 μL MCH-MCHR1-G_{i1} complex at the concentration of 5 mg/mL or 3 μL SNAP-94847-bound MCHR1-mBRIL-Fab1B3-Glue complex at the concentration of 3 mg/mL was applied to a glow-discharged holey Ni-Ti alloy grid (ANTcryo, M01, Au300 R1.2/1.3). The grid was blotted and frozen in liquid ethane using Vitrobot Mark IV (Thermo Fischer Scientific). The grids were imaged on a 300 kV Titan Krios electron microscope (Thermo Fischer Scientific) equipped with Gatan K3 Summit direct electron detector and an energy filter. Data were collected at the magnification of 81,000× at a pixel size of 0.535 Å in super-resolution mode using the EPU software. Image stacks were recorded in 32 frames at a total dose of 55 $e^-/Å^2$ with the defocus range from -2.2 to -1.2 μm. A total of 4,901 movies for MCH-MCHR1-G_{i1} complex and 5713 movies for SNAP-94847-bound MCHR1-mBRIL-Fab1B3-Glue complex were collected.

Cryo-EM data processing

For MCH-MCHR1-G_{i1} complex, 4,901 movies were subjected to CryoSPARC[5] and processed with Patch motion correction and Patch CTF estimation. Exposures with tolerable CTF fit resolution and total motion distance were selected for further processing. Blob picker was used to pick particles from a small subset of micrographs for creation of 2D templates. Particles were picked from the whole dataset by Template picker using these 2D templates. After 2D classification, six 3D classes were generated by Ab-initio Reconstruction. Particles from 4 classes were further classified by Heterogeneous Refinement. Then two classes with acceptable quality were pooled and re-classified into 6 classes using Ab-initio Reconstruction and Heterogeneous Refinement. Four classes with 871,951, 771,833, 588,396, and 500,329 particles were individually processed by Non-uniform Refinement[6] and improved by Local Refinement with a customized global mask. The final maps reach the nominal resolution of 2.61 Å, 2.65 Å, 2.78 Å, and 2.81 Å at a Fourier shell correlation (FSC) threshold of 0.143. Estimation of local resolution and local filtering of the maps were performed in CryoSPARC.

For SNAP-94847-MCHR1-mBRIL complex, 5713 movies were subjected to CryoSPARC and

processed with MotionCor2 and CTFFIND4. Exposures with tolerable CTF fit resolution and total motion distance were selected for further processing. Blob picker was used to pick particles from a small subset of micrographs for creation of 2D templates. Particles were picked from the whole dataset by Template picker using these 2D templates. After 2D classification, four 3D classes were generated by Ab-initio Reconstruction. Then all particles were classified into these four classes by Heterogeneous Refinement. Three classes with acceptable quality were pooled and re-classified into 4 classes using Ab-initio Reconstruction and Heterogeneous Refinement. Two classes with 305,549 and 268,193 particles were individually processed by Non-uniform Refinement. The final maps reached the nominal resolution of 3.33 Å and 3.43 Å at a Fourier shell correlation (FSC) threshold of 0.143. Estimation of local resolution of the maps were performed in CryoSPARC.

Model building and validation

For MCH-MCHR1-G_{i1}-scFv16 complex, the initial model of MCHR1 was generated by Alphafold[7]. Coordinates of G_{i1}-scFv16 was derived from the μOR-G_{i1} complex (PDB ID: 6DDE). MCH was manually built in Coot according to the density.

For SNAP-94847-MCHR1-mBRIL-Fab1B3-Glue complex, the initial model of MCHR1-mBRIL was also generated by Alphafold. Coordinates of mBRIL and Fab1B3 was derived from the crystal structure of BRIL in complex with Fab1B3 (PDB ID: 8J7E). Coordinates of E3 and K3 helices were derived from the structure of the E3/K3 coiled-coil (PDB ID: 1U0I). Coordinates of NbFab were derived from an NbFab-contained cryo-EM structure (PDB ID: 7PHP). Coordinates of ALFA tag and NbALFA were derived from the crystal structure of NbALFA bound to ALFA tag peptide (PDB ID: 6I2G). Coordinates and geometry restraints of SNAP-94847 were generated using eLBOW in Phenix.

The models were fitted into the EM map and combined using UCSF Chimera[8]. Then the model was corrected by manual adjustment in Coot[9] and refined by Real-space refinement in Phenix[10]. Model statistics were calculated by MolProbity[11] and provided in Supplementary Table 1.

G protein-dissociation assay

Function of wild-type and mutant MCHR1 was measured using the TRUPATH biosensors as previously described[12]. HEK293T cells were distributed into six-well plates at a density of 1.2×10^6 cells per well and incubated for 8 h at 37 °C. A plasmid mixture of 0.5 μg wild-type or mutant MCHR1, 0.5 μg $G\alpha_{i1}$-RLuc8, 0.5 μg $G\beta_3$, 0.5 μg GFP2-$G\gamma_9$ was co-transfected into HEK293T cells using Lipofectamine 3000 (Thermo Fisher Scientific). After 40 h, cells were harvested, washed with HBSS (Hank's Balanced Salt Solution), and resuspended in 800 μL BRET buffer (HBSS supplemented with 25 mM HEPES pH 7.4 and 0.1% BSA). Cells were divided into white-wall white-bottom 96-well plates at the density of 100,000 cells per well. Then the luciferase substrate coelenterazine 400a at 5 μM working concentration was added and the plates were incubated at room temperature for 5 minutes. Cells were stimulated with MCH at different final concentrations before the plates were incubated for another 5 minutes at room temperature. The BRET signal was

recorded by SpectraMax iD5 (Molecular Devices) and calculated as the ratio of light emission at 515 nm (GFP2)/410 nm (RLuc8). Data were baseline-corrected with the ligand-free control and curves were calculated by a three-parameter logistic function. Data from three independent experiments were used for analysis.

To measure the activity of antagonist SNAP-94847, we carried out the same procedures as those for MCHR1-mediated $G\alpha_{i1}$ dissociation from $G\beta_1\gamma_2$, except that HEK293T cells were pre-treated with different concentrations of SNAP-94847 dissolved in assay buffer from 10^{-11} M to 10^{-4} M and incubated for 10 min. After that, 10 μM MCH were added to the wells and incubated for 5 min. The BRET signal was recorded by SpectraMax iD5 (Molecular Devices) and calculated as the ratio of light emission at 515 nm (GFP2)/410 nm (RLuc8). Data were baseline-corrected with the antagonist-free control and curves were calculated by a three-parameter logistic function. Data from three independent experiments were used for analysis.

Cell-surface expression analysis

Cell-surface expression of wild-type MCHR1 and mutants was measured by a fluorescence-activated cell sorting (FACS) assay. HEK293T cells were seeded in 24-well plates at the density of 2×10^5 cells per well before transfected with 0.5 μg plasmid encoding FLAG-tagged wild-type MCHR1 or mutants using Lipofectamine 3000. After 42 h, cells were collected and resuspended in HBSS. 20 μL cells were incubated with 20 μL anti-FLAG M2-FITC antibody (Sigma Aldrich) diluted in TBS buffer containing 20 mM Tris pH 7.5, 150 mM NaCl, and 4% (w/v) BSA at 4 °C for 20 min. 160 μL HBSS buffer supplemented with 5 mM HEPES pH 7.4 was added after incubation. The fluorescence was measured on CytoFLEX (Beckman). The gate was set by FSC/SSC thresholds to define single cells. Surface expression level was evaluated by mean fluorescence intensity and normalized to the mock and wild-type group. Data from three independent experiments were used for analysis.

Molecular dynamics (MD) simulations

A 400ns molecular dynamics simulation was performed using GROMACS version 2024.1 to examine the interaction between MCHR1 and its antagonist, SNAP-94847. Prior to the simulation, SWISS-MODEL was employed for homology modeling to complete the MCHR1 structure by filling in missing residues. The missing residues that were not elucidated clearly by cryo-EM were added using the sequence recorded in Uniprot ID Q99705. Another simulation was conducted using MCHR1 without the BRIL and C-tag sequences. The H++ server was then utilized to ensure proper residue protonation states at pH 7.0. Ligand forcefield preparation involved using antechamber in AmberTools with the AM1-BCC charge model for atomic point charge calculations. The ligand library was generated using the General AMBER force field 2 (GAFF2). For protein residues, the ff19SB forcefield was selected. Given MCHR1's nature as a membrane protein, a lipid bilayer was prepared using PACKMOL-Memgen. This lipid bilayer comprised phosphatidylcholine, 1-palmitoyl-2-oleoyl-sn-glycero-3-phosphocholine, and cholesterol in a 7:3 ratio. The water layer above the membrane or protein was set to 15Å in the z-axis, using the TIP3P water model. The

system also included 0.15M KCl. Energy minimization was conducted for 50,000 steps with a 0.002ps time step using the steep integrator. System equilibration lasted 300ps with a 0.002ps time step, fixing the protein's heavy atoms and ligand atoms. The subsequent 400ns molecular dynamics simulation used a 0.002ps time step. Both equilibration and simulation phases employed v-rescale thermostat and Berendsen pressure coupling, with the system temperature set to 300K. The bonds between hydrogens and heavy atoms were also constrained.

Molecular docking

To investigate the binding modes of other antagonists of MCHR1, we performed molecular docking studies using AutoDock Vina[13]. MCHR1 from the cryo-EM structure of SNAP-94847-MCHR1 was used as the receptor. Coordinates of different antagonists were downloaded from PubChem. Coordinates of the receptor and the ligands were processed by AutoDockTools using default settings. The docking box was a 15~18 Å cube centered on SNAP-94847 in the cryo-EM structure. No flexible residues of the receptor were defined. Binding poses were selected according to binding energy and visual inspection.

Data availability

The atomic coordinates of MCH-MCHR1-G_{i1} complex in different states (T1, T2, L1, and L2) have been deposited in the Protein Data Bank (PDB) under accession codes 8WWK, 8WWL, 8WWN, and 8WWM, respectively. The EM maps of MCH-MCHR1-G_{i1} complex have been deposited in the Electron Microscopy Data Bank (EMBD) under accession codes EMD-37891, EMD-37892, EMD-37894, and EMD-37893, respectively. The atomic coordinates of SNAP-94847-bound MCHR1-mBRIL complex in S1 and S2 states have been deposited in the Protein Data Bank (PDB) under accession codes 8YNS and 8YNT, respectively. The EM maps of SNAP-94847-bound MCHR1-mBRIL complex have been deposited in the Electron Microscopy Data Bank (EMBD) under accession codes EMD-39429 and EMD-39430, respectively.

References

1 Duan, J. *et al.* Cryo-EM structure of an activated VIP1 receptor-G protein complex revealed by a NanoBiT tethering strategy. *Nat Commun* **11**, 4121, doi:10.1038/s41467-020-17933-8 (2020).

2 Qi, X. *et al.* Cryo-EM structure of oxysterol-bound human Smoothened coupled to a heterotrimeric Gi. *Nature* **571**, 279-283, doi:10.1038/s41586-019-1286-0 (2019).

3 Koehl, A. *et al.* Structure of the μ-opioid receptor-Gi protein complex. *Nature* **558**, 547-552, doi:10.1038/s41586-018-0219-7 (2018).

4 Guo, Q. *et al.* A method for structure determination of GPCRs in various states. *Nat Chem Biol* **20**, 74-82, doi:10.1038/s41589-023-01389-0 (2024).

5 Punjani, A., Rubinstein, J. L., Fleet, D. J. & Brubaker, M. A. cryoSPARC: algorithms for rapid unsupervised cryo-EM structure determination. *Nat Methods* **14**, 290-296, doi:10.1038/nmeth.4169 (2017).

6 Punjani, A., Zhang, H. & Fleet, D. J. Non-uniform refinement: adaptive regularization improves single-particle cryo-EM reconstruction. *Nat Methods* **17**, 1214-1221, doi:10.1038/s41592-020-00990-8 (2020).

7 Jumper, J. *et al.* Highly accurate protein structure prediction with AlphaFold. *Nature* **596**, 583-589, doi:10.1038/s41586-021-03819-2 (2021).

8 Pettersen, E. F. *et al.* UCSF Chimera--a visualization system for exploratory research and analysis. *J Comput Chem* **25**, 1605-1612, doi:10.1002/jcc.20084 (2004).

9 Emsley, P. & Cowtan, K. Coot: model-building tools for molecular graphics. *Acta Crystallographica Section D* **60**, 2126-2132, doi:doi:10.1107/S0907444904019158 (2004).

10 Liebschner, D. *et al.* Macromolecular structure determination using X-rays, neutrons and electrons: recent developments in Phenix. *Acta Crystallogr D Struct Biol* **75**, 861-877, doi:10.1107/S2059798319011471 (2019).

11 Williams, C. J. *et al.* MolProbity: More and better reference data for improved all-atom structure validation. *Protein Sci* **27**, 293-315, doi:10.1002/pro.3330 (2018).

12 Olsen, R. H. J. *et al.* TRUPATH, an open-source biosensor platform for interrogating the GPCR transducerome. *Nat Chem Biol* **16**, 841-849, doi:10.1038/s41589-020-0535-8 (2020).

13 Trott, O. & Olson, A. J. AutoDock Vina: improving the speed and accuracy of docking with a new scoring function, efficient optimization, and multithreading. *J Comput Chem* **31**, 455-

461, doi:10.1002/jcc.21334 (2010).
