# Supplementary material for: Structural insights into physiological activation and antagonism of melanin-concentrating hormone receptor MCHR1
Source: Cell Discov. 2024 Nov 30;10:124. doi: 10.1038/s41421-024-00754-0 (PMC11608246; doi:10.1038/s41421-024-00754-0)
Supplement: Supplementary file 3 — Supplementary tables [file 41421_2024_754_MOESM3_ESM.pdf]

Supplementary Tables for

Structural insights into physiological activation and antagonism of melanin-concentrating hormone receptor MCHR1

Xiaofan Ye, Guibing Liu, Xiu Li, Binbin He, Yuyong Tao, Jiasheng Guan, Yuguang Mu, Haiping Liu, Weimin Gong

Table of Contents

Supplementary Tables 2

Supplementary Table 1 | Cryo-EM data collection, refinement, and validation statistics 2

Supplementary Table 2 | pEC50 values, Emax and the expression level of mutations of MCH-binding pocket and ICL1 in MCHR1 3

Supplementary Table 3 | pEC50 values, Emax and the expression level of mutations of SNAP-94847-binding pocket in MCHR1 4

Supplementary Tables

Supplementary Table 1 | Cryo-EM data collection, refinement, and validation statistics

	**MCH-MCHR1-G_{i1} T1 state (EMD-37891) (PDB 8WWK)**	**MCH-MCHR1-G_{i1} T2 state (EMD-37892) (PDB 8WWL)**	**MCH-MCHR1-G_{i1} L1 state (EMD-37894) (PDB 8WWN)**	**MCH-MCHR1-G_{i1} L2 state (EMD-37893) (PDB 8WWM)**	**SNAP-94847-MCHR1 S1 state (EMD-39429) (PDB 8YNS)**	**SNAP-94847-MCHR1 S2 state (EMD-39430) (PDB 8YNT)**
Magnification	81,000	81,000	81,000	81,000	81,000	81,000
Voltage (kV)	300	300	300	300	300	300
Electron exposure ($e^-/Å^2$)	55	55	55	55	55	55
Defocus range (μm)	-2.2 to -1.2	-2.2 to -1.2	-2.2 to -1.2	-2.2 to -1.2	-2.2 to -1.2	-2.2 to -1.2
Pixel size (Å)	1.07	1.07	1.07	1.07	1.07	1.07
Symmetry imposed	C1	C1	C1	C1	C1	C1
Initial particle images (no.)	8,634,044	8,634,044	8,634,044	8,634,044	2,912,457	2,912,457
Final particle images (no.)	871,951	588,396	771,833	500,329	305,549	268,193
Map resolution (Å)	2.61	2.78	2.65	2.81	3.33	3.43
FSC threshold	0.143	0.143	0.143	0.143	0.143	0.143
Map resolution range (Å)	2.5-5.0	2.5-5.0	2.5-5.0	2.5-5.0	2.5-5.0	2.5-5.0
**Refinement**						
Initial model used (PDB code)	6DDE	6DDE	6DDE	6DDE	-	-
Model resolution (Å)	2.71	2.87	2.75	2.96	3.42	3.59
FSC threshold	0.5	0.5	0.5	0.5	0.5	0.5
Model composition						
Non-hydrogen atoms	8967	8964	8960	8963	7296	7420
Protein residues	1160	1160	1160	1160	958	974
Ligands	0	0	0	0	1	1
B factors ($Å^2$)						
Protein	32.30	39.99	30.12	62.41	89.47	86.85
Ligand	-	-	-	-	96.64	106.19
R.m.s. deviations						
Bond lengths (Å)	0.002	0.002	0.002	0.004	0.002	0.002
Bond angles (°)	0.392	0.438	0.464	0.501	0.396	0.389
Validation						
MolProbity score	1.25	1.32	1.26	1.32	1.23	1.58
Clashscore	4.78	5.86	4.96	5.91	3.96	5.25
Poor Rotamers (%)	0.72	0.83	0.62	0.93	1.16	1.65
Ramachandran plot						
Favored (%)	99.21	98.43	98.78	98.25	98.42	97.30
Allowed (%)	0.79	1.57	1.22	1.75	1.58	2.70
Disallowed (%)	0	0	0	0	0	0

Supplementary Table 2 | pEC50 values, Emax and the expression level of mutations of MCH-binding pocket and ICL1 in MCHR1

Mutant	pEC50±SEM	Emax （%WT）±SEM	Expression （%WT）±SEM
		MCH	
WT	7.12±0.17	100	100
$K139^{ICL1}A$	NA	NA	124±7
$K139^{ICL1}E$	NA	NA	126±13
$L140^{ICL1}A$	6.99±0.21	71±2	119±12
$L140^{ICL1}E$	7.15±0.36	52±2	70±10
$C143^{ICL1}A$	6.83±0.21	92±4	143±13
$C143^{ICL1}R$	6.98±0.15	108±2	88±20
$M168^{2.60}A$	6.57±0.35	53±4	101±4
$D192^{3.32}A$	NA	NA	65±10
$Q196^{3.36}A$	NA	NA	139±19
$I254^{ECL2}A$	NA	NA	77±9
$F256^{ECL2}A$	5.89±0.41	72±19	68±8
$F277^{5.38}A$	NA	NA	113±19
$W338^{6.48}A$	NA	NA	90±5
$Y341^{6.51}A$	NA	NA	61±14
$Q345^{6.55}A$	6.97±0.47	57±4	91±21
$Q348^{6.58}A$	7.49±0.10	112±1	120±9
$Y362^{7.35}A$	NA	NA	63±13
$I366^{7.39}A$	NA	NA	96±7
$Y370^{7.42}A$	NA	NA	80±6

The response data was normalized by WT receptor within each individual experiment. Data from three independent experiments are presented as mean ± S.E.M. NA, no activity.

Supplementary Table 3 | pEC50 values, Emax and the expression level of mutations of SNAP-94847-binding pocket in MCHR1

Mutant	pEC50±SEM	Emax （%WT）±SEM	Expression （%WT）± SEM
		MCH	
WT	7.12±0.19	100	100
F116$^{1.39}$A	7.63±0.17	61±1	98±9
D158$^{2.50}$A	NA	NA	133±7
F161$^{2.53}$A	7.10±0.35	77±4	109±15
M168$^{2.60}$A	7.45±0.41	55±2	156±25
S195$^{3.35}$A	6.87±0.28	80±4	132±9
S373$^{7.46}$A	7.21±0.21	83±2	154±21
		MCH (10 μM)+SNAP-94847	
WT	6.27±0.36	100	100
F116$^{1.39}$A	7.23±0.41	81±1	98±9
D158$^{2.50}$A	6.64±1.03	27±2	133±7
F161$^{2.53}$A	6.85±0.36	93±1	109±15
M168$^{2.60}$A	5.20±0.34	73±8	156±25
S195$^{3.35}$A	6.43±0.21	147±2	132±9
S373$^{7.46}$A	6.25±0.17	149±6	154±21

The response data was normalized by WT receptor within each individual experiment. Data from three independent experiments are presented as mean ± S.E.M. NA, no activity.
